# Arabic translation, cultural adaptation, and preliminary validation of the International Olympic Committee Medical Report of Injury and Illness form

**DOI:** 10.1186/s40621-026-00678-1

**Published:** 2026-04-13

**Authors:** Azad M. Jan, Doaa Aljasser, Fahad Bin Radhyan, Abdulrahman I. Alaqil

**Affiliations:** 1 Research and Innovation, Leaders Development Institute, Ministry of Sports, Riyadh, Saudi Arabia; 2 Epidemiology and Biostatistics Section, Health Sciences Research Center, Princess Nourah Bint Abdulrahman University, Riyadh, Saudi Arabia; 3 Department of Physical Education, College of General Studies, King Fahd University of Petroleum and Minerals, Dhahran, Saudi Arabia; 4 Department of Physical Education, College of Education, King Faisal University, Al-Ahsa, 31982 Saudi Arabia

**Keywords:** Cultural adaptation, Injury surveillance, Sports medicine, Saudi Arabia, Arabic translation, IOC Medical Report, Content validity, Face Validity

## Abstract

**Background:**

Surveillance of injuries and illnesses is crucial to safeguarding athletes’ health and performance. Obtaining reliable, comparable data requires the application of standardized definitions and methodologies.

**Aim:**

This study sought to (i) translate the International Olympic Committee (IOC) Medical Report of Injury and Illness form from English to Arabic, (ii) culturally adjust it for use with Arabic speakers, and (iii) test it for use among Arabic-speaking athletes.

**Methods:**

The original English version of the Medical Report of Injury form was translated into Arabic using internationally recognized methods and cross-cultural adaptation guidelines. This process included forward-backwards translation, expert review, and cognitive interviews. The participants (*N* = 19) comprised Saudi Arabian athletes with previous injuries, coaches, and sports medicine professionals. The preliminary content validity was evaluated using the Item-Level Content Validity Index (I-CVI) and Scale-Level Content Validity Index (S-CVI/Ave). Face validity was assessed using cognitive interviews with 15 of the 19 participants.

**Results:**

The I-CVI scores for all 14 items ranged from 0.86 to 1.00; the S-CVI/Ave score was 0.96, both of which indicate good agreement content validity. A total of 13 items were rated “highly suitable” (score = 4); one item (“no return to sport possible”) attracted a score of 3. Cognitive interviews confirmed that all items were clear and culturally appropriate for the Arabic participants. Minor wording changes were made to some items based on participant feedback.

**Conclusion:**

The Arabic version of the IOC Medical Report of Injury and Illness form demonstrated high preliminary content and face validity. Therefore, this version is deemed suitable for use with Arabic-speaking athletes for standardized injury and illness reporting. Adopting the Arabic version of the form is expected to improve the accuracy and comparability of epidemiological data and may foster its integration into digital injury surveillance systems.

**Supplementary Information:**

The online version contains supplementary material available at 10.1186/s40621-026-00678-1.

## Introduction

Participating in sports offers significant benefits for physical and mental health. However, it also increases the risk of injuries and illnesses, the severity of which depends on both intrinsic (age, sex, anatomical differences) and extrinsic (type of sport, training intensity, equipment, playing surface) factors [1, 2]. Gathering accurate surveillance data on sports-related injuries and illnesses is vital for assessing risks, identifying patterns, and implementing evidence-based mitigation strategies [3–5].

In Saudi Arabia, the rapid growth of participation in organized sports has heightened the need for robust epidemiological data on sports-related injuries and illnesses [6]. Unfortunately, research on this topic in this context remains inconsistent and scarce. Extant studies are limited in quality and scope, drawing on retrospective hospital databases or focusing exclusively on particular demographic groups, such as elite athletes [7–12]. Furthermore, extant studies have poor methodological consistency, especially in how injuries are defined, classified, and reported, which makes comparisons and pooled analysis challenging [5]. Notably, most existing studies disproportionately focus on male athletes, despite the significant rise in female participation in sports [6].

To address the above shortcomings in standardized injury and illness surveillance among athletes, the International Olympic Committee’s (IOC) 2020 consensus statement sets out methodological protocols for the collection and reporting of sports-related epidemiological data [5]. The mainstay of these guidelines, the IOC Medical Report of Injury or Illness form, uses a standardized format to capture consistent data across athletes participating in international sporting events [5, 13, 14]. To support the tool’s use among athletes from diverse linguistic and cultural backgrounds, the IOC explicitly recommends that it be translated and culturally adapted to serve these populations [5]. While translation ensures the tool is understandable to speakers of different languages, it must also be culturally adapted to preserve the conceptual meaning and practical applicability of each item. This ensures that the tool will cater to the nuances of the various healthcare and sports-related practices across international populations of athletes, including communication styles and contextual realities. This enables the collection of accurate, relevant, and comparable data from athlete populations within each respective local setting [15].

Despite the IOC’s recommendation, there is, to date, no validated Arabic version of the IOC Medical Report of Injury or Illness form. To address this gap, this study had two aims. First, to translate, culturally adapt, and produce an Arabic version of the IOC Medical Report of Injury or Illness with preliminary content and face validity. Second, to prepare the Arabic version for electronic implementation and enhance usability by enabling real-time data collection and integration into national/international surveillance systems. Ultimately, this study provides researchers, clinicians, and sports medicine practitioners in Arabic-speaking regions with a standardized, culturally relevant research instrument to collect accurate and comparable surveillance data on sports-related injuries and illnesses.

## Method

### Study design and participants

A cross-sectional, convenience sampling methodology was used. The sample (*N* = 19) consisted of native Arabic-speaking coaches and healthcare professionals involved in sports settings. Participants were recruited from various sports clubs and academic institutions in Saudi Arabia between 17th February and 27th April 2024. The inclusion criteria were: (i) participants fluent in Arabic, and (ii) active participants in sports or sports healthcare-related roles. The sample was selected to reflect the intended user hierarchy of Arabic version of the IOC Medical Report of Injury or Illness form. Healthcare professionals (e.g., physicians and physiotherapists) represent the primary end-users responsible for documenting and reporting injuries and illnesses during sporting events, while coaches may serve as secondary users in certain applied settings. Athletes were not included, as the form is designed to be completed by trained personnel rather than by athletes themselves. Therefore, Prior personal experience of sporting injury was not required, as the evaluation focused on clarity, terminology, and usability from a professional perspective. The sample was considered appropriate for the objectives of this preliminary evaluation. Ethical approval was secured from the Institutional Review Board at Princess Nourah Bint Abdulrahman University (IRB: 24–0186). The Declaration of Helsinki was adhered to, and all participants provided written informed consent.

### Instrument

This section outlines the comprehensive procedures for translating and culturally adapting the English version of the IOC Medical Report of Injury or Illness into Arabic for use by Arabic-speaking injured athletes, coaches, and sports healthcare professionals. The original version of the IOC Medical Report of Injury or Illness was developed as a standardized assessment tool to systematically record and monitor injuries and illnesses incurred by athletes during the Olympic Games or related events. The original version is publicly available, recommended by the International Olympic Committee (IOC), and available only in English [5]. The tool contains 14 items intended to gather data on injury and/or illness suffered by athletes. The assessment tool is relatively brief and should take 5–10 min to complete [5].

### Translation and cultural adaptation process

Established guidelines for the cross-cultural adaptation of such tools, as recommended in [15] and previously implemented in [16, 17], were followed.

#### Translation

Forward translation into Arabic was independently performed by two proficient bilingual (Arabic and English) translators with expertise in medical terminology. This step ensured the translated version of the tool from English to Arabic was culturally and linguistically accurate.

Next, back-translation of the Arabic version of the tool (detailed above) was performed by two independent bilingual translators with no prior exposure to the original English version. These translators were blind to the study’s objectives. The translators back-translated the tool from Arabic into English. This step was carried out to ensure conceptual equivalence between the two versions and identify any inconsistencies or divergence in meaning that may have occurred during the forward translation.

Next, two sports medicine and medical translation experts reviewed the Arabic version and the back-translated English version and compared them with the original English version of the tool. This step aimed to evaluate and confirm the semantic, conceptual, and contextual equivalence between the English and the newly produced Arabic versions. Any identified discrepancies were highlighted, discussed, and resolved through consensually. This step ensured that the final Arabic version of the tool was both accurate and culturally appropriate for Arabic speakers.

### Content validity

Content validity was evaluated by two sports science experts by applying the Content Validity Index (I-CVI) [18–21]. These experts were independent from the translation process. The 14 items were separately rated on a 4-point scale (1 = not relevant, 4 = highly relevant). Scores 3 and 4 were coded as “1” (relevant), while scores of 1 and 2 were coded as “0” (not relevant). The I-CVI was calculated as the proportion of the two experts who rated an item as relevant. The S-CVI/Ave was calculated as the average of all I-CVI values. Only two evaluators were employed due to resource constraints and the limited availability of Arabic-speaking experts with specific expertise in sports injury and illness surveillance and familiarity with the IOC Medical Report of Injury or Illness form. While this approach is acceptable for preliminary evaluation, CVI estimates derived from such a small panel should be interpreted with caution, as they may be unstable and prone to inflated agreement. Therefore, the findings should be considered as supporting preliminary content validity only and do not constitute full validation of the instrument. The commonly used threshold of ≥ 0.80 was achieved; however, this should be interpreted cautiously given the small number of expert raters. Future studies should consider using larger expert panels and applying modified kappa statistics to adjust for chance agreement and strengthen the robustness of content validity assessment (see Table 1).

**Table 1:** item-level content Validity Index (I-CVI)

Items	I-CVI for relevance	I-CVI for clarity
Q1 Competition or training	1	1
Q2 Mode of onset	1	1
Q3 Injury mechanism	1	1
Q4 Injury type	1	1
Q5 Organ system	1	1
Q6 Time – lost in sports due to injury/illness	1	1
Q7 No return to sport possible	1	1

### Face validity

Face validity was assessed by asking a sample (*N* = 15) of participants working as coaches and healthcare professionals in Saudi Arabia to complete an initial iteration of the Arabic version of the tool. Participants were asked to provide feedback on the tool’s clarity, terminology, and cultural appropriateness. Semi-structured cognitive interviews featuring a combination of open- and closed-ended questions were used to gather these data [22]. Participant feedback was noted by the interviewer. All interviews were conducted in Arabic by a sports medicine expert. Following each round of interviews, recurring suggested changes to the tool were reviewed, synthesized, and incorporated iteratively into the Arabic version of the tool (Table 3). Participants completed the Arabic form and provided feedback on clarity, terminology, and cultural suitability. The process continued until data saturation was reached, defined as the point at which no new feedback emerged from participants. All suggested modifications were reviewed and incorporated (Table 3).

### Statistical analysis

Research Electronic Data Capture (REDCap 7.3.6- © 2022 Vanderbilt University) was used for data collection and management. To assess the tool’s validity, participants were asked to complete it by reporting previous sporting injuries. JMP version 18 was used to assess content validity; the Item-level content validity index (I-CVI) [20, 21] was calculated for each item, respectively. The Scale-Level Content Validity Index (S-CVI/Ave) was calculated as the average of I-CVI across all 14 items.

## Result

Forward translation into Arabic was performed by two proficient Arabic < > English translators experienced in medical terminology in both languages. This process highlighted several linguistic issues with the items, some of which required rephrasing to use the most appropriate Arabic terms that matched the English meanings.

The first iteration of the translated Arabic version of the tool was reviewed and revised by the above-mentioned expert translators to ensure that all items accurately conveyed the original English meanings in appropriate Arabic terms. All decisions on the most suitable Arabic phrasing of the items were made after consideration of the linguistic context of each item in the English version.

Two sports science specialists rated the 14 items in the Arabic version of the tool using the Content Validity Index guidelines outlined above. A total of 13 items attracted a score of 4, indicating high suitability; only one item (no return to sport possible) attracted a score of 3 (low suitability).

As shown in Table 2, of the 19 participants, 57.9% (*n* = 11) were male; 42.1% (*n* = 8) were female. Most participants were from Saudi Arabia (78.9%); the remainder were from Sudan (10.5%), Yemen (5.3%), and Syria (5.3%). The largest proportion of participants were football coaches (36.8%), followed by fitness coaches (31.6%) and physiotherapists (21.0%). One participant was a tennis coach (5.3%); one was a medical doctor (5.3%).

**Table 2:** Participants Demographics N (%) (*n* = 19):

	Frequency (%)
**Sex**	
Male	11 (57.9)
Female	8 (42.1)
**Country**	
Saudi Arabia	15 (78.9)
Yemin	1 (5.3)
Sudan	2 (10.5)
Syria	1 (5.3)
**Occupation**	
Fitness Coach	6 (31.6)
Football Coach	7 (36.8)
Tennis Coach	1 (5.3)
Medical Doctor	1 (5.3)
Physiotherapist	4 (21)

A total of 15 participants matched the inclusion criteria. Cognitive interviews were conducted to ensure face validity. The final Arabic version of the tool was modified based on participants’ feedback, as shown in Table 3.

**Table 3:** Changes provided by the study participants of the Medical Report of Injury or Illness questionnaire (*n* = 15)

Item in English	Item in Arabic	Modification
**Competition or training:** Competition. Training. Peri-competition (e.g. warm-up, cool- down).	خلال المنافسة أم التدريب: منافسة. تدريب. ما قبل المنافسة (مثل الاحماء او الاطالة).	هل حدثت الاصابة خلال المنافسة أم التدريب؟ منافسة. تدريب. تدريبات مصاحبة للمنافسة (مثل الاحماء او الاطالة).
**Mode of onset:** Sudden after acute trauma. Sudden but no acute trauma. Gradual. Mixed.	طريقة بداية الاصابة: مفاجئة بعد حدوث صدمة شديدة. مفاجئة من غير حدوث صدمة شديدة. تدريجية. متنوع/خليط مما سبق.	طريقة بداية الاصابة: مفاجئة بعد حدوث اصطدام شديد. مفاجئة من غير حدوث اصطدام شديد تدريجي. متنوع/خليط مما سبق.
**Injury mechanism**: No identifiable. Single event (repetitive transfer of energy, overuse) Acute non-contact trauma. Direct contact with another athlete. Following contact with another athlete (e.g. fall after a push). Direct contact with an object (e.g. ball, wall, ground, i.e. slipped and fell). Following contact with an object.	**آلية حدوث الاصابة**: لا يوجد آلية محددة للإصابة. صدمة من دون اتصال مباشر. اثناء اتصال مباشر مع لاعب اخر. اصابة بعد اتصال مباشر مع لاعب اخر. اثناء اتصال مباشر مع جسم ما. اصابة بعد اتصال مباشر مع جسم ما.	**آلية حدوث الاصابة**: لا يوجد آلية محددة للإصابة. صدمة من دون اتصال مباشر. اثناء احتكاك مباشر مع لاعب اخر. اصابة بعد احتكاك مباشر مع لاعب اخر. اثناء احتكاك مباشر مع جسم ما. اصابة بعد احتكاك مباشر مع جسم ما.
**Injury type:** Avascular necrosis. Physis injury. Muscle compartment syndrome. Bursitis. Synovitis. Stump injury.	**نوع الاصابة**: النخر اللاوعائي. اصابة صفيحة النمو. متلازمة المقصورة العضلية. التهاب جرابي/التجويف الكيسي. التهاب الغشاء الزلالي. اصابة طرف مبتور.	**نوع الاصابة**: النخر اللاوعائي/Avascular necrosis اصابة صفيحة النمو Physis injury/ متلازمة المقصورة العضلية Muscle/compartment syndrome التهاب جرابي/التجويف الكيسي/Bursitis. التهاب الغشاء الزلالي Synovitis/ اصابة طرف مبتور Stump injury/
**Organ system**: Thermoregulatory system.	**الجهاز العضوي**: تنظيم حراري.	**الجهاز العضوي**: تنظيم حراري/Thermoregulatory system
**Time – lost in sports due to injury/illness**: No. Yes.	**خسارة الوقت في الرياضة بسبب الاصابة أو المرض**: لا. نعم.	**هل توقف/ت الاعب/ة عن المنافسة أو التدريب بسبب الاصابة أو المرض؟** لا. نعم.
**No return to sport possible**: Fatality. Permanent disability. Other reasons.	**السبب في حال ان العودة الى ممارسة الرياضة غير ممكنة**: الوفاة. اعاقة دائمة. اسباب اخرى.	**السبب في حال ان العودة الى ممارسة الرياضة غير ممكنة**: الوفاة. اعاقة دائمة. اسباب اخرى.

## Discussion

This study resulted in the successful translation and cultural adaptation of the IOC Medical Report of Injury and Illness form from English to Arabic. It succeeded in creating a standardized, linguistically accurate, culturally appropriate, and contextually relevant tool with preliminary content and face validity for use among Arabic-speaking sports professionals and participants. Semantic, idiomatic, and conceptual equivalence between the original English and Arabic versions was achieved through the rigorous methodology detailed above. The Arabic version of the tool aligns with internationally recognized guidelines for cross-cultural adaptation of such research tools. The results indicated high preliminary content and face validity, underscoring its reliability for injury and illness surveillance in sports medicine among Arabic populations [4, 6].

The content validity scores (I-CVI range = 0.86–1.00; S-CVI/Ave = 0.96) indicate high agreement among the experts. However, given the small number of raters, these findings should be interpreted cautiously and are considered to provide preliminary support for content validity rather than definitive evidence of validity. Additionally, these findings compare favorably with previous sports science cross-cultural validation studies in terms of similar agreement levels [16, 17, 21, 23–26]. The slightly lower rating for a single item (“no return to sport possible”) can be attributed to cultural differences in interpreting medical and rehabilitation outcomes. In Arabic, the meanings of permanent disability and fatality subtly vary across dialects and medical contexts; these warrants further refinement of these terms in future iterations of the Arabic version of the tool. That said, the high levels of agreement across the remaining 13 items highlight the accuracy and clarity of the Arabic version of the tool, indicating that the tool retained both its original intent and clinical utility.

The present findings concur with prior findings on the necessity of using standardized and culturally adapted research instruments in sports medicine research. Similar efforts to validate Arabic versions of English-language tools for data collection on physical activity, training load, and sedentary behavior have emphasized the importance of cultural equivalence and linguistic accuracy [16, 17, 21, 24, 27]. Extant sports injury surveillance studies in Saudi Arabia and the Middle East have limited comparability and generalizability as they often rely on unstandardized or retrospective hospital data [6–12, 28, 29]. To address this shortcoming in the research, the present study’s Arabic version of the IOC Medical Report provides a consistent, standardized data collection instrument with high agreement content and face validity across Arabic athletes, sports teams, institutions, and nations. This tool allows researchers to compare such data internationally, as recommended by the IOC [5, 13, 14].

Culturally adapting the tool to cater to Arabic-speaking populations was vital to ensuring its usability and accuracy [23]. Terms that required careful linguistic calibration to reflect the meanings of professional medical terminology and everyday sports language (as encapsulated in the English-language items) in the Arabic version included *acute trauma*,* direct contact*, and *peri-competition*. The forward-and-back-translation and collaborative review process conducted by the bilingual translators and sports medicine specialists ensured that Arabic version maintained conceptual integrity with the original English version and were applicable to Arabic-speaking populations. The statistical analysis (I-CVI scores for all 14 items: 0.86–1.00.86.00; S-CVI/Ave score: 0.96) indicates high agreement among experts and provides preliminary support for the content validity of the Arabic version of the instrument. However, these findings should be interpreted cautiously due to the limited number of exert raters and do not constitute full validation. The instrument may be suitable for initial use in research and practice settings; however, further psychometric evaluation is required before confirming its broader applicability for standardized surveillance of sports-related injuries and illnesses among Arabic-speaking populations.

The Arabic version of the Medical Report of Injury and Illness form presented in the current study offers several benefits for illness and injury surveillance, contributing to the prevention, monitoring, and research of injuries and illness among athletes from Arabic-speaking populations. First, it provides a culturally and linguistically standardized data collection tool for Arabic-speaking populations across sports clubs, universities, and healthcare institutions throughout the Middle East and North Africa. Second, the tool will enable the digital integration of illness and injury surveillance data from Arabic-speaking populations. This will facilitate real-time data entry, automated aggregation, and longitudinal monitoring to support national databases and international collaborations, such as with the IOC. Sports policymakers and governing bodies will be able to leverage such data to identify high-risk sporting activities, efficiently allocate resources, and develop targeted interventions and prevention programs. Third, the inclusion of female participants in this study also addresses gender disparities in sports injury surveillance to ensure that the tool is inclusive and representative of all members of athlete populations [8, 29]. Finally, the proposed instrument offers content and face validity to ensure the robustness of data it gathers on the intended concepts.

One key strength of this study is its comprehensive adherence to established forward-and-back translation methods, cross-cultural adaptation protocols, and its use of both expert and participant feedback to ensure the instrument offers good agreement face and content validity [15, 18–21]. The inclusion of professionals from relevant disciplines (coaches, physiotherapists, and physicians) broadened the perspectives considered in creating the tool.

Despite its merits, the current study is subject to five main limitations. First, the sample size was small (*N* = 19), although acceptable for preliminary content and face validity assessment, is insufficient for full psychometric validation. Therefore, the findings should be interpreted as part of an initial adaptation phase rather than a comprehensive validation of the instrument. Additionally, the sample is not representative of all Arabic-speaking athletes’ limiting the generalizability of results. Further evaluation of the tool using larger and more diverse samples is required to establish its broader applicability and full psychometric properties. Participants were selected using convenience sampling rather than purposeful saturation, consisted predominantly of coaches and healthcare professionals, and underrepresented athletes, who are the primary end users of the form. As such, the results cannot be generalized to all Arabic athletes or applied across different competitive levels. Second, although the preliminary content and face validity of the tool were established, no additional psychometric testing (i.e., test–retest reliability and inter-rater reliability) was conducted. Specifically, reliability analyses, construct validity, criterion validity, and measurement error assessments were not performed. Therefore, the current findings should be interpreted as an initial adaptation phase rather than full validation. Future studies using larger, multi-center samples are needed to comprehensively evaluate the instrument’s psychometric properties. Third, as mentioned, while the current Arabic version of the IOC tool enables data collection to provide valuable preliminary insights on Arabic-speaking athletes, we acknowledge the potential bias this may have introduced to CVI scores and recommend future researchers recruit a larger, more diverse expert panel. Fourth, content validity was assessed by only two experts. Although the CVI can be calculated with a small number of raters, estimates derived from two expert lack robustness and are particularly sensitive to individual judgment. The small panel size may increase the likelihood of inflated agreement and limit the interpretability of the indices, as it is difficult to distinguish true consensus from chance agreement. Consequently, the reported CVI values should be interpreted with caution and considered indicative of preliminary content validity only. Future studies should involve larger and more diverse expert panels and consider complementary approaches, such as modified kappa statistics, to provide more stable and reliable estimates. Finally, the tool does not account for dialectal diversity across Arabic-speaking countries. This shortcoming could be addressed by conducting research to formulate alternative versions of the tool adapted to athletes who speak a range of Arabic dialects. However, it is important to note that Modern Standard Arabic was used in the current study, and efforts were made to ensure dialect-neutral phrasing through careful translation and expert review to enhance cross-regional applicability. Future work could benefit from expanding the tool’s content and face validity to include additional Arabic-speaking countries to assess its cross-dialectal and cultural consistency. Also, to help determine its usability in real-world conditions and facilitate continuous refinement, the tool could be deployed to collect surveillance data on injuries and illnesses from Arabic-speaking participants at live sporting events. Researchers are also encouraged to explore digital implementation strategies, such as integrating the Arabic version of the IOC Medical Report into electronic health record systems and monitoring applications for athletes, coaches, and medical professionals. Finally, developing and implementing educational workshops for coaches, medical practitioners, and data managers to standardize their use of the tool will likely enhance the accuracy of the surveillance data collected and promote its broader adoption.

The Arabic version of the IOC Medical Report of Injury and Illness form proposed in this paper is based on expert translation, cultural adaptation, and preliminary content and face validity assessment. It represents the preliminary stages of instrument development for sports injury documentation and surveillance in Arabic-speaking populations rather than full validation. The tool bridges a critical gap in standardized illness and injury surveillance data reporting and contributes to global efforts to achieve harmonized epidemiological data collection [3, 5, 6, 13, 14]. It is hoped that by ensuring linguistic precision, cultural relevance, and methodological rigor, the Arabic version of the tool will empower practitioners, policymakers, and researchers to improve athlete safety by guiding the development of preventive interventions and strengthening the evidence base for sports medicine across the Arab world.

## Conclusion

This study provides an initial adaptation and preliminary phase to ensure the content and face validity of the proposed Arabic version of the IOC Medical Report of Injury and Illness form. The tool intended to enhance the consistency and comparability of sports injury and illness surveillance data across Arabic-speaking populations. The rigorous translation process followed international protocols to ensure linguistic and conceptual accuracy. The high CVI scores indicate strong agreement among experts regarding item clarity and relevance; however, these findings should be interpreted within the context of a limited expert panel. This work provides an important first step toward addressing gap in sport injury surveillance in Arabic-speaking contexts. Future research is required to establish additional psychometric properties, including reliability, construct validity, and measurement error, using larger and more diverse samples. with continued validation, the instrument has the potential to contribute to improved surveillance practices and alignment with international IOC reporting standards.

## Supplementary Information

Supplementary Material 1

## Data Availability

The datasets used and/or analyzed during the current study available from the corresponding author on reasonable request.

## References

[1] Bahr R, Krosshaug T. Understanding injury mechanisms: a key component of preventing injuries in sport. Br J Sports Med. 2005;39:324–9. 10.1136/bjsm.2005.018341. 15911600 10.1136/bjsm.2005.018341 PMC1725226

[2] Hopkins WG, Marshall SW, Quarrie KL, Hume PA. Risk factors and risk statistics for sports injuries. Clin J Sport Med Off J Can Acad Sport Med. 2007;17:208–10. 10.1097/JSM.0b013e3180592a68. 10.1097/JSM.0b013e3180592a68 17513914

[3] Ekegren CL, Gabbe BJ, Finch CF. Sports injury surveillance systems: a review of methods and data quality. Sports Med. 2016;46:49–65. 10.1007/s40279-015-0410-z. 10.1007/s40279-015-0410-z 26423419

[4] de Loës M. Exposure data. Why are they needed? Sports Med. 1997;24:172–5. 10.2165/00007256-199724030-00005. 9327532 10.2165/00007256-199724030-00005

[5] International Olympic Committee Injury and Illness Epidemiology Consensus Group, Bahr R, Clarsen B, Derman W, Dvorak J, Emery CA, et al. International Olympic Committee consensus statement: methods for recording and reporting of epidemiological data on injury and illness in sports 2020 (including the STROBE extension for sports injury and illness surveillance (STROBE-SIIS)). Orthop J Sports Med. 2020;8:2325967120902908. 10.1177/2325967120902908. 32118084 10.1177/2325967120902908 PMC7029549

[6] Al-Hazzaa HM, Albawardi NM. Obesity, lifestyle behaviors, and dietary habits of Saudi adolescents living in Riyadh (ATLS-2 project): revisited after a ten-year period. Life Basel Switz. 2021;11:1078. 10.3390/life11101078. 10.3390/life11101078 PMC8537999 34685449

[7] Almansour L, Mohammad WS, Elsais W, Alonazi A, Alyahya D, Almansour L. Unveiling the knee injury landscape: a comprehensive study of youth male football players in the Central Region of Saudi Arabia. Appl Sci. 2024. 10.3390/app14093895.

[8] Al Attar WSA, Bizzini M, Alkabkabi F, Alshamrani N, Alarifi S, Alzahrani H, et al. Effectiveness of the FIFA 11 + referees injury prevention program in reducing injury rates in male amateur soccer referees. Scand J Med Sci Sports. 2021;31:1774–81. 10.1111/sms.13983. 33914964 10.1111/sms.13983

[9] Shobian A, Hamdi MS, Bakhamees AS. Epidemiology of sports-related injuries among athletes in Jeddah, Saudi Arabia. Egypt J Hosp Med Pan Arab Leag Continuous Med Educ. 2017;69:2607–13. 10.12816/0042236.

[10] Almaawi A, Awwad W, Bamugaddam A, Alasheikh M, Muaddi M, Almutair O, et al. Prevalence of knee injuries among male college students in Riyadh, Kingdom of Saudi Arabia. J Orthop Surg Res. 2020;15:126. 10.1186/s13018-020-01638-1. 32238180 10.1186/s13018-020-01638-1 PMC7110648

[11] Al Attar WSA, Bizzini M, Alzahrani H, Alarifi S, Ghulam H, Alyami M, et al. The FIFA 11 + kids injury prevention program reduces injury rates among male children soccer players: a clustered randomized controlled trial. Sports Health. 2023;15:397–409. 10.1177/19417381221109224. 35903029 10.1177/19417381221109224 PMC10170224

[12] Almutawa M, Scott M, George KP, Drust B. The incidence and nature of injuries sustained on grass and 3rd generation artificial turf: a pilot study in elite Saudi National Team footballers. Phys Ther Sport Off J Assoc Chart Physiother Sports Med. 2014;15:47–52. 10.1016/j.ptsp.2013.02.004. 10.1016/j.ptsp.2013.02.004 23791754

[13] Junge A, Engebretsen L, Alonso JM, Renström P, Mountjoy M, Aubry M, et al. Injury surveillance in multi-sport events: the International Olympic Committee approach. Br J Sports Med. 2008;42:413–21. 10.1136/bjsm.2008.046631. 18390916 10.1136/bjsm.2008.046631

[14] Jacobsson J, Timpka T, Ekberg J, Kowalski J, Nilsson S, Renström P. Design of a protocol for large-scale epidemiological studies in individual sports: the Swedish Athletics injury study. Br J Sports Med. 2010;44:1106–11. 10.1136/bjsm.2009.067678. 20484318 10.1136/bjsm.2009.067678

[15] Beaton DE, Bombardier C, Guillemin F, Ferraz MB. Guidelines for the process of cross-cultural adaptation of self-report measures. Spine. 2000;25:3186–91. 11124735 10.1097/00007632-200012150-00014

[16] Alaqil AI, Gupta N, Alothman SA, Al-Hazzaa HM, Stamatakis E, Cruz B, et al. Arabic translation and cultural adaptation of sedentary behavior, dietary habits, and preclinical mobility limitation questionnaires: a cognitive interview study. PLoS One. 2023;18:e0286375. 10.1371/journal.pone.0286375. 37307255 10.1371/journal.pone.0286375 PMC10259774

[17] Bursais AK. Arabic translation and cultural adaptation of a training load and player monitoring in high-level football questionnaire: a cognitive interview study. PLoS One. 2024;19:e0302006. 10.1371/journal.pone.0302006. 38630762 10.1371/journal.pone.0302006 PMC11023223

[18] Zamanzadeh V, Ghahramanian A, Rassouli M, Abbaszadeh A, Alavi-Majd H, Nikanfar A-R. Design and implementation content validity study: development of an instrument for measuring patient-centered communication. J Caring Sci. 2015;4:165–78. 10.15171/jcs.2015.017. 26161370 10.15171/jcs.2015.017 PMC4484991

[19] Haynes SN, Richard DCS, Kubany ES, American Psychological Association. Content validity in psychological assessment: a functional approach to concepts and methods. Psychol Assess. 1995;7:238–47. 10.1037/1040-3590.7.3.238.

[20] Polit DF, Beck CT. The content validity index: are you sure you know what’s being reported? Critique and recommendations. Res Nurs Health. 2006;29:489–97. 10.1002/nur.20147. 16977646 10.1002/nur.20147

[21] Alhaji JH, Alshuwaier GO, Alharbi NS, Alaqil AI, BinSultan NM, Alonazi WB. Translation and validation of the Arabic version of the capability assessment for diet and activity (CADA) questionnaire in Saudi University employed women. Int J Environ Res Public Health. 2021. 10.3390/ijerph18126246. 34207752 10.3390/ijerph18126246 PMC8296046

[22] Willis GB. Analysis of the Cognitive Interview in Questionnaire Design. Oxford University Press; 2015.

[23] Gjersing L, Caplehorn JR, Clausen T. Cross-cultural adaptation of research instruments: language, setting, time and statistical considerations. BMC Med Res Methodol. 2010;10:13. 10.1186/1471-2288-10-13. 20144247 10.1186/1471-2288-10-13 PMC2831007

[24] Musaiger AO, Al-Mannai M, Tayyem R, Al-Lalla O, Ali EYA, Kalam F, et al. Perceived barriers to healthy eating and physical activity among adolescents in seven Arab countries: a cross-cultural study. Sci World J. 2013;2013:232164. 10.1155/2013/232164. 10.1155/2013/232164 PMC3848306 24348144

[25] Wiesinger GF, Nuhr M, Quittan M, Ebenbichler G, Wölfl G, Fialka-Moser V. Cross-cultural adaptation of the Roland-Morris questionnaire for German-speaking patients with low back pain. Spine. 1999;24:1099–103. 10361659 10.1097/00007632-199906010-00009

[26] Sousa VD, Rojjanasrirat W. Translation, adaptation and validation of instruments or scales for use in cross-cultural health care research: a clear and user-friendly guideline: validation of instruments or scales. J Eval Clin Pract. 2011;17:268–74. 10.1111/j.1365-2753.2010.01434.x. 20874835 10.1111/j.1365-2753.2010.01434.x

[27] AlHadi AN, AlAteeq DA, Al-Sharif E, Bawazeer HM, Alanazi H, AlShomrani AT, et al. An arabic translation, reliability, and validation of patient health questionnaire in a Saudi sample. Ann Gen Psychiatry. 2017;16:32. 10.1186/s12991-017-0155-1. 28878812 10.1186/s12991-017-0155-1 PMC5585978

[28] Al Attar WSA, Faude O, Bizzini M, Alarifi S, Alzahrani H, Almalki RS, et al. The FIFA 11 + shoulder injury prevention program was effective in reducing upper extremity injuries among soccer goalkeepers: a randomized controlled trial. Am J Sports Med. 2021;49:2293–300. 10.1177/03635465211021828. 34138672 10.1177/03635465211021828

[29] Sadat-Ali M, Sankaran-Kutty M. Sports injuries in Saudi Arabia. Br J Sports Med. 1985;19:28–9. 10.1136/bjsm.19.1.28. 3995225 10.1136/bjsm.19.1.28 PMC1478232

