# Supplementary material for: Arabic translation, cultural adaptation, and preliminary validation of the International Olympic Committee Medical Report of Injury and Illness form
Source: Inj Epidemiol. 2026 Apr 13;13:30. doi: 10.1186/s40621-026-00678-1 (PMC13134105; doi:10.1186/s40621-026-00678-1)
Supplement: Supplementary file 1 — Supplementary Material 1 [file 40621_2026_678_MOESM1_ESM.pdf]

التقرير الطبي اليومي للإصابات والأمراض

الدولة **تاريخ التقرير**

تم تعبئة التقرير من قبل: **الاسم:** معلومات التواصل

الرجاء تسجيل المعلومات لكلٍ من: **(1)** جميع الإصابات الرياضية (2) وجميع الأمراض التي يعاني منها الرياضيين في فريقك، الجديدة منها أو المتكررة أو ما إذا كانت تفاقماً لإصابة مسبقة مستقرة أو مرض مسبق مستقر وذلك أثناء <اذكر اسم البطولة> بغض النظر عن الآثار المترتبة للغياب عن المنافسة أو التدريب. تعد جميع المعلومات المزودة سرية وسيتم التعامل معها بناء على ذلك.

1. الإصابات – مثال للمصطلحات والرموز (راجع الصفحة الخلفية)

العمر	الجنس	الرياضة والمنافسة الرياضية	تاريخ الإصابة	المنافسة / التدريب	الرمز	رمز بداية الإصابة	الرمز الجديد
22	**ذكر/أنثى**	لعبة الشاري	21 يوليو	سباق السرعة	2	1	~~4~~
آلية حدوث الإصابة	الرمز	منطقة الإصابة في الجسم	الرمز	نوع الإصابة	الرمز	خسارة الوقت	المدة
انزلق وسقط	5	الكاحل	17	التواء	10	نعم / ~~لا~~	28 يوماً

العمر	الجنس **ذكر/أنثى**	الرياضة والمنافسة الرياضية	تاريخ الإصابة	المنافسة / التدريب	الرمز	رمز بداية الإصابة	الرمز الجديد
آلية حدوث الإصابة	الرمز	منطقة الإصابة في الجسم	الرمز	نوع الإصابة	الرمز	خسارة الوقت نعم / لا	المدة

العمر	الجنس **ذكر/أنثى**	الرياضة والمنافسة الرياضية	تاريخ الإصابة	المنافسة / التدريب	الرمز	رمز بداية الإصابة	الرمز الجديد
آلية حدوث الإصابة	الرمز	منطقة الإصابة في الجسم	الرمز	نوع الإصابة	الرمز	خسارة الوقت نعم / لا	المدة

العمر	الجنس **ذكر/أنثى**	الرياضة والمنافسة الرياضية	تاريخ الإصابة	المنافسة / التدريب	الرمز	رمز بداية الإصابة	الرمز الجديد
آلية حدوث الإصابة	الرمز	منطقة الإصابة في الجسم	الرمز	نوع الإصابة	الرمز	خسارة الوقت نعم / لا	المدة

2. الأمراض – مثال للمصطلحات والرموز (راجع الصفحة الخلفية)

العمر	الجنس	الرياضة والمنافسة الرياضية	تاريخ بداية المرض	الجهاز العضوي/منطقة العضو المصاب	الرمز
27	**ذكر/أنثى**	ألعاب القوى، القفز بالزانة	24 يوليو	الجهاز التنفسي	13
مسببات المرض		الرمز	رمز المرض إن كان جديد أم متكرر أم متفاقم	خسارة الوقت	المدة
بيئي – غير متعلق بالتمرين		3	1	نعم / ~~لا~~	2

العمر	الجنس **ذكر/أنثى**	الرياضة والمنافسة الرياضية	تاريخ بداية المرض	الجهاز العضوي/منطقة العضو المصاب	الرمز
مسببات المرض		الرمز	رمز المرض إن كان جديد أم متكرر أم متفاقم	خسارة الوقت نعم / لا	المدة

العمر	الجنس **ذكر/أنثى**	الرياضة والمنافسة الرياضية	تاريخ بداية المرض	الجهاز العضوي/منطقة العضو المصاب	الرمز
مسببات المرض		الرمز	رمز المرض إن كان جديد أم متكرر أم متفاقم	خسارة الوقت نعم / لا	المدة

العمر	الجنس **ذكر/أنثى**	الرياضة والمنافسة الرياضية	تاريخ بداية المرض	الجهاز العضوي/منطقة العضو المصاب	الرمز
مسببات المرض		الرمز	رمز المرض إن كان جديد أم متكرر أم متفاقم	خسارة الوقت نعم / لا	المدة

العمر	الجنس **ذكر/أنثى**	الرياضة والمنافسة الرياضية	تاريخ بداية المرض	الجهاز العضوي/منطقة العضو المصاب	الرمز
مسببات المرض		الرمز	رمز المرض إن كان جديد أم متكرر أم متفاقم	خسارة الوقت نعم / لا	المدة

الرجاء استخدام نسخ إضافية في حال لم يتسع التقرير للإبلاغ عن جميع الإصابات أو الأمراض

☐ لا توجد إصابة أو مرض جديدين في أحد من لاعبي الفريق اليوم.

للإصابات (هي: تلف في الأنسجة أو خلل في الوظائف الحركية الاعتيادية نتيجة للمشاركة في الرياضة، أو ناجم عن طاقة حركية متسارعة أو متكررة)

المنافسات أو التدريب
1. خلال المنافسة، الرجاء ذكر اسم المنافسة الرياضية
2. تدريب
3. قبل المنافسة (فترة الإحماء أو الإطالة)

طريقة بداية الإصابة
1. مفاجئة بعد حدوث اصطدام شديد
2. مفاجئة من غير حدوث اصطدام
3. تدريجية
4. متنوع / خليط مما سبق

آلية حدوث الإصابة
1. لا يوجد آلية محددة للإصابة
2. صدمة من دون احتكاك مباشر
3. أثناء احتكاك مباشر مع لاعب آخر
4. إصابة بعد احتكاك مباشر مع لاعب آخر
5. أثناء احتكاك مباشر مع جسم ما
6. إصابة بعد احتكاك مباشر مع جسم ما

منطقة الإصابة في الجسم
1. الرأس / الوجه
2. الرقبة / الفقرات العنقية
3. الصدر (بما في ذلك الأعضاء الداخلية)
4. الفقرات الصدرية / أعلى الظهر
5. الفقرات القطنية / الأرداف
6. البطن (بما في ذلك الأعضاء الداخلية)
7. الكتف
8. العضد
9. المرفق / الكوع
10. الساعد
11. الرسغ / المعصم
12. اليد
13. الورك / الأربية
14. الفخذ
15. الركبة
16. الساق / وتر العرقوب /أخيليس
17. الكاحل
18. القدم

نوع الإصابة
1. ارتجاج في المخ / إصابة في الدماغ
2. إصابة في الحبل الشوكي
3. إصابة في عصب طرفي
4. كسور عظام
5. كسور إجهاد العظام
6. كدمة في العظم
7. نخر لا وعائي / avascular necrosis
8. إصابة صفيحة النمو / physis injury
9. إصابة غضروف
10. التواء مفصل / تمزق رباط
11. عدم استقرار المفصل
12. تمزق وتر
13. اعتلال الأوتار
14. إجهاد عضلي أو تمزق في العضلة
15. كدمة عضلية
16. متلازمة المقصورة العضلية / muscle compartment syndrome
17. جرح عميق
18. خدش
19. كدمة / رضة (سطحية)
20. التهاب في المفصل
21. التهاب جرابي / التجويف الكيسي /bursitis
22. التهاب الغشاء الزلالي / synovitis
23. تلف الأوعية الدموية
24. إصابة طرف مبتور / stump injury
25. صدمة في الأعضاء الداخلية
26. إصابة غير معروفة / غير محددة

للأمراض (هي شكوى أو اضطراب غير متعلق بإصابة)

الجهاز العضوي
1. قلب وأوعية دموية
2. جلدي
3. أسنان
4. غدد صماء
5. هضمي
6. بولي / تناسلي
7. دموي
8. هيكلي عضلي
9. عصبي
10. بصري
11. سمعي
12. نفسي
13. تنفسي
14. تنظيم حراري / thermoregulatory system
15. غير معروف / غير محدد

المسببات المرضية
1. حساسية
2. بيئية – متعلقة بالتمرين
3. بيئي – غير متعلق بالتمرين
4. مناعي / التهاب
5. عدوى
6. ورم
7. أيضية / تغذوية
8. وعاء دموي
9. حالة انتكاسية أو مزمنة
10. شذوذ نموي / نشوئي
11. تسمم / متعلق بعقار طبي
12. غير محدد / غير معروف

للإصابات والأمراض

الرياضة والمنافسة الرياضية
الرجاء تسجيل معلومات الرياضة (مثل ألعاب القوى) وحدد المنافسة الرياضية (القفز بالزانة) إن وجد

إصابة جديدة أم متكررة أم متفاقمة
1. حديثة ظهرت أثناء البطولة
2. متكررة بعد الشفاء التام والعودة لممارسة الرياضة بشكل كامل
3. متفاقمة لحالة مستقرة لم يتم التشافي منها
4. غير معلوم أو غير محدد

خسارة الوقت في الرياضة بسبب الإصابة أو المرض
لا استمر اللاعب في التدريب أو المنافسة، حتى لو كان ذلك على غير المستوى المعتاد (بالنسبة للمدة والشدة والأداء)

نعم لم يكمل اللاعب التدريب أو المنافسة عند حدوث الإصابة **أو** لم يستطيع المشاركة في اللعب لاحقاً

المدة مشاركة في اللعب غير اعتيادية / أداء محدود في الرياضة نتيجة الإصابة أو المرض (بالأيام)
الرجاء إعطاء تقدير لعدد الأيام التي لن يستطع اللاعب/ة اللعب فيها المشاركة في اللعب أو التدريب كالمعتاد، وذلك باحتساب اليوم الذي يلي اليوم الذي حدثت فيه الإصابة باليوم 1.

في حال كان من **غير المتوقع** رجوع اللاعب للرياضة بعد الإصابة أو المرض، الرجاء ذكر السبب : و = وفاة، ع = إعاقة دائمة ، أ = أسباب أخرى

التقرير الطبي للإصابة والمرض

تاريخ التقرير:

الفريق | رقم اللاعب/ة | تاريخ الإصابة / المرض

+

للإصابات

خلال المنافسة أم التدريب

☐ منافسة ☐ تدريب ☐ ما قبل المنافسة (مثل الإحماء او الإطالة)

طريقة بداية الإصابة

☐ مفاجئة بعد حدوث اصطدام شديد ☐ مفاجئة من غير حدوث اصطدام ☐ تدريجية ☐ متنوع / خليط مما سبق

آلية حدوث الإصابة (قد تحتوي كل فئة على فئة فرعية وفقاً لغرض المتابعة)

☐ لا يوجد آلية محددة للإصابة ☐ أثناء احتكاك مباشر مع لاعب آخر ☐ أثناء احتكاك مباشر مع جسم ما
☐ اصابة من دون احتكاك مباشر ☐ إصابة بعد احتكاك مباشر مع لاعب آخر ☐ إصابة بعد احتكاك مباشر مع جسم ما

منطقة الإصابة في الجسم (قد تحتوي كل فئة على فئة فرعية وفقاً لغرض المتابعة)

☐ الرأس / الوجه
☐ الرقبة / الفقرات العنقية
☐ الصدر (بما في ذلك الأعضاء الداخلية)
☐ الفقرات الصدرية / أعلى الظهر
☐ الفقرات القطنية / الأرداف
☐ البطن (بما في ذلك الأعضاء الداخلية)
☐ الكتف
☐ العضد
☐ المرفق / الكوع
☐ الساعد
☐ الرسغ / المعصم
☐ اليد
☐ الورك / الأربية
☐ الفخذ
☐ الركبة
☐ الساق / وتر العرقوب/أخيليس
☐ الكاحل
☐ القدم

نوع الإصابة

☐ ارتجاج في المخ / إصابة في الدماغ
☐ إصابة في الحبل الشوكي
☐ إصابة في عصب طرفي
☐ كسور عظام
☐ كسور إجهاد العظام
☐ كدمة في العظم
☐ نخر لا وعائي / avascular necrosis
☐ إصابة صفيحة النمو / physis injury
☐ إصابة غضروف
☐ التواء مفصل / تمزق رباط
☐ عدم استقرار المفصل
☐ تمزق وتر
☐ اعتلال الأوتار
☐ إجهاد عضلي أو تمزق في العضلة
☐ كدمة عضلية
☐ متلازمة المقصورة العضلية / muscle compartment syndrome
☐ جرح عميق
☐ خدش
☐ كدمة / رضة (سطحية)
☐ التهاب في المفصل
☐ التهاب جرابي / التجويف الكيسي /bursitis
☐ التهاب الغشاء الزلالي / synovitis
☐ تلف الأوعية الدموية
☐ إصابة طرف مبتور / stump injury
☐ صدمة في الأعضاء الداخلية
☐ إصابة غير معروفة / غير محددة

للأمراض

الجهاز العضوي

☐ قلب وأوعية دموية
☐ جلدي
☐ أسنان
☐ غدد صماء
☐ هضمي
☐ بولي / تناسلي
☐ دموي
☐ هيكلي عضلي
☐ عصبي
☐ بصري
☐ سمعي
☐ نفسي
☐ تنفسي
☐ تنظيم حراري / thermoregulatory system
☐ غير معروف / غير محدد

المسببات المرضية

☐ حساسية
☐ بيئية – متعلقة بالتمرين
☐ بيئي – غير متعلق بالتمرين
☐ مناعي / التهاب
☐ عدوى
☐ ورم
☐ أيضية / تغذوية
☐ وعاء دموي
☐ حالة انتكاسية أو مزمنة
☐ شذوذ نموي / نشوئي
☐ تسمم / متعلق بعقار طبي
☐ غير محدد / غير معروف

للإصابات والأمراض

جديدة أم متكررة أم متفاقمة

☐ جديدة ☐ متكررة بعد الشفاء التام والعودة لممارسة الرياضة بشكل كامل ☐ غير معلوم أو غير محدد
☐ متفاقمة لحالة مستقرة لم يتم التشافي منها

خسارة الوقت في الرياضة بسبب الإصابة أو المرض

☐ لا ☐ نعم

تاريخ العودة الكامل للتمرين المعتاد والمنافسة ____________ (اليوم/الشهر/السنة)

السبب في حال أن العودة إلى ممارسة الرياضة **غير ممكنة**:

☐ الوفاة ☐ إعاقة دائمة ☐ أسباب أخرى ____________
